# Statistical methods for assessing the effects of *de novo* variants on birth defects

**DOI:** 10.1186/s40246-024-00590-z

**Published:** 2024-03-14

**Authors:** Yuhan Xie, Ruoxuan Wu, Hongyu Li, Weilai Dong, Geyu Zhou, Hongyu Zhao

**Affiliations:** 1 Department of Biostatistics, Yale School of Public Health, 60 College Street, New Haven, CT 06520 USA; 2 Department of Genetics, Yale School of Medicine, New Haven, CT 06520 USA

**Keywords:** *De novo* variants, Birth defects, Integrative analysis

## Abstract

With the development of next-generation sequencing technology, *de novo* variants (DNVs) with deleterious effects can be identified and investigated for their effects on birth defects such as congenital heart disease (CHD). However, statistical power is still limited for such studies because of the small sample size due to the high cost of recruiting and sequencing samples and the low occurrence of DNVs. DNV analysis is further complicated by genetic heterogeneity across diseased individuals. Therefore, it is critical to jointly analyze DNVs with other types of genomic/biological information to improve statistical power to identify genes associated with birth defects. In this review, we discuss the general workflow, recent developments in statistical methods, and future directions for DNV analysis.

## Introduction

Birth defects are structural changes present at birth that can affect one part or several parts of the body, such as the heart and brain [1]. They pose significant challenges for both individual health and public health. Learning about the causes of birth defects is crucial for improving the quality of support and resources to help individuals and families affected. It is estimated that around 240,000 infants globally do not survive past their first 28 days every year due to birth defects, with these conditions also leading to the deaths of an additional 170,000 children aged one month to five years [2]. There are several possible causes of birth defects, including genetic changes, adverse reactions to medications, exposure to substances or chemicals, or complications during pregnancy. It is estimated that about 20% of birth defects are caused by genetic factors [3]. There are in general three general categories of genetic causes: chromosomal abnormalities, single-gene defects, and multifactorial influences [4]. Efforts have been made to identify genetic causes of birth defects [5]. The role of rare variants in disease genetics has been unraveled with the development of whole exome sequencing (WES) and whole genome sequencing (WGS) technologies. In this review, we exclusively focus on the statistical methods that can be applied to *de novo* single nucleotide variants (SNVs) and small insertions/deletions (indels). They are referred to as *de novo* variants (DNVs), within the purview of birth defects research. Compared to other rare variants, DNVs represent an extreme case, given their very low occurrence and large effect size. On average, an individual may carry approximately 100 DNVs in the genome, with about 1 variant affecting the exome [6–9]. DNVs have been considered strong supporting evidence for pathogenicity based on the American College of Medical Genetics and Genomics classification guidelines and provide important insights into the genetic cause of diseases [10].

Mounting evidence has pinpointed the importance of conducting DNV analyses to identify risk genes for birth defects such as congenital heart disease (CHD), congenital diaphragmatic hernia (CDH), orofacial cleft (OFC) [9, 11–15], and some early onset neurodevelopmental disorders such as autism [16–19]. In the study of WES data from 2,645 proband-parent trios by the Pediatric Cardiac Genomics Consortium (PCGC), Jin et al. found that DNVs accounted for 8% of cases and inferred that DNVs in about 440 genes contributed to CHD [13]. In the largest genetic exploration of coding DNVs affected by nonsyndromic OFCs to date, Bishop et al. analyzed the contribution of coding DNVs from WGS to OFC risks in 756 proband-parent trios and identified multiple promising genes that had not been reported before, such as *ZFHX3* and *ZFHX4* [9]. In 2021, Qiao et al. conducted an analysis of coding DNVs sequenced from 827 CDH proband-parent trios. They confirmed an overall enrichment of damaging DNVs in constrained genes (ExAC [20] pLI score > 0.5) and identified *LONP1* and *ALYREF* as candidate CDH-associated genes with a false discovery rate (FDR) of 0.05 [15]. These results shed new insights into the disease etiology of birth defects, call upon the application of statistical methods that can analyze the enrichment of DNV in other birth defect cohorts, and motivate the development of novel statistical methods to improve the power of identifying genes associated with birth defects.

In this review, we first summarize the general workflow of conducting DNV analysis, including data preprocessing, mutation rate calculation, and DNV enrichment analysis. Next, we introduce several integrative statistical methods that can further incorporate DNVs with other types of variants or biological information to boost the power of risk gene identification (Table 1). In addition, we discuss several potential future directions for DNV analysis in birth defects.

**Table 1:** Summary of statistical methods reviewed in this paper

Name	Estimation	Inference		Inputs of Additional Information	DNV Data Used in Real Data Application	Software Link	Reference	
denovolyzeR	-	Poisson exact test		-	1,078 autism spectrum disorder (ASD) trios	https://denovolyzer.org/	[8]	
DeNovoWEST	-	Simulation-based test		-	31,058 developmental disorder (DD) trios	https://github.com/HurlesGroupSanger/DeNovoWEST	[32]	
TADA	Empirical Bayes method	Bayesian false discovery rate (FDR) and *p*-value from permutation test		Inherited variants	932 ASD trios	http://www.compgen.pitt.edu/TADA/TADA_homepage.htm	[17]	
extTADA	Fully Bayesian framework	Bayesian FDR		Inherited variants	1,077 schizophrenia (SCZ) trios; 4,293 DD trios; 1,022 intellectual disability (ID) trios; 4,122 ASD trios; 356 epilepsy trios	https://github.com/hoangtn/extTADA	[40]	
TADA-R	Empirical Bayes method	*p*-value from permutation test		Inherited variants	2,645 congenital heart disease (CHD) trios; 1,789 unaffected control trios	https://github.com/limo936/TADA-R	[41]	
fitDNM	-	Score test		Functional annotations	264 epileptic encephalopathy (EE) trios; 151 severe ID trios; 354 SCZ trios; 948 ASD trios	https://github.com/TNTurnerLab/fitDNM	[42]	
VARPRISM	Maximum Likelihood Estimation (MLE)-based method	Likelihood ratio test		Functional annotations	2,508 ASD trios	https://hufflab.org/software/VARPRISM/	[44]	
TADA-A	MLE-based method	Bayesian FDR		Functional annotations	314 ASD trios	https://github.com/TADA-A/TADA-A	[46]	
HeartENN	Deep learning-based method	Binomial test		Noncoding annotations	749 CHD trios	https://github.com/FunctionLab/HeartENN-models	[49]	
mTADA	Fully Bayesian framework	Bayesian FDR		*De novo* variants (DNVs) from another trait	356 EE trios; 5,122 ASD trios; 4,293 DD trios; 1,012 ID trios; 1,077 SCZ trios; 1,213 CHD trios	https://github.com/hoangtn/mTADA	[50]	
M-DATA	Expectation-Maximization algorithm	Joint local false discovery rate (Jlfdr)		DNVs from another trait and functional annotations	2,645 CHD trios; 5,623 ASD trios	https://github.com/JustinaXie/MDATA	[51]	
DAWN	Empirical Bayes method	Bayesian FDR		Interaction network	932 ASD trios	http://www.compgen.pitt.edu/DAWN/DAWN_homepage.htm	[53, 54]	
N-DATA	Empirical Bayes method	Bayesian FDR		Interaction network	2,645 CHD trios	https://github.com/JustinaXie/NDATA	[55]	
VBASS	Variational inference	Bayesian FDR		Gene expression	2,645 CHD trios; 16,616 ASD trios	https://github.com/ShenLab/VBASS	[56]	
EncoreDNM	Monte Carlo MLE	Likelihood ratio test		DNVs from another trait	31,058 DD trios; 6,430 ASD trios; 2,722 SCZ trios; 2,645 CHD trios; 820 ID trios; 484 Tourette disorder trios; 264 EE trios; 232 congenital hydrocephalus trios; 1,789 unaffected control trios	https://github.com/ghm17/EncoreDNM	[52]	

## General workflow of DNV analysis

**Fig. 1:**
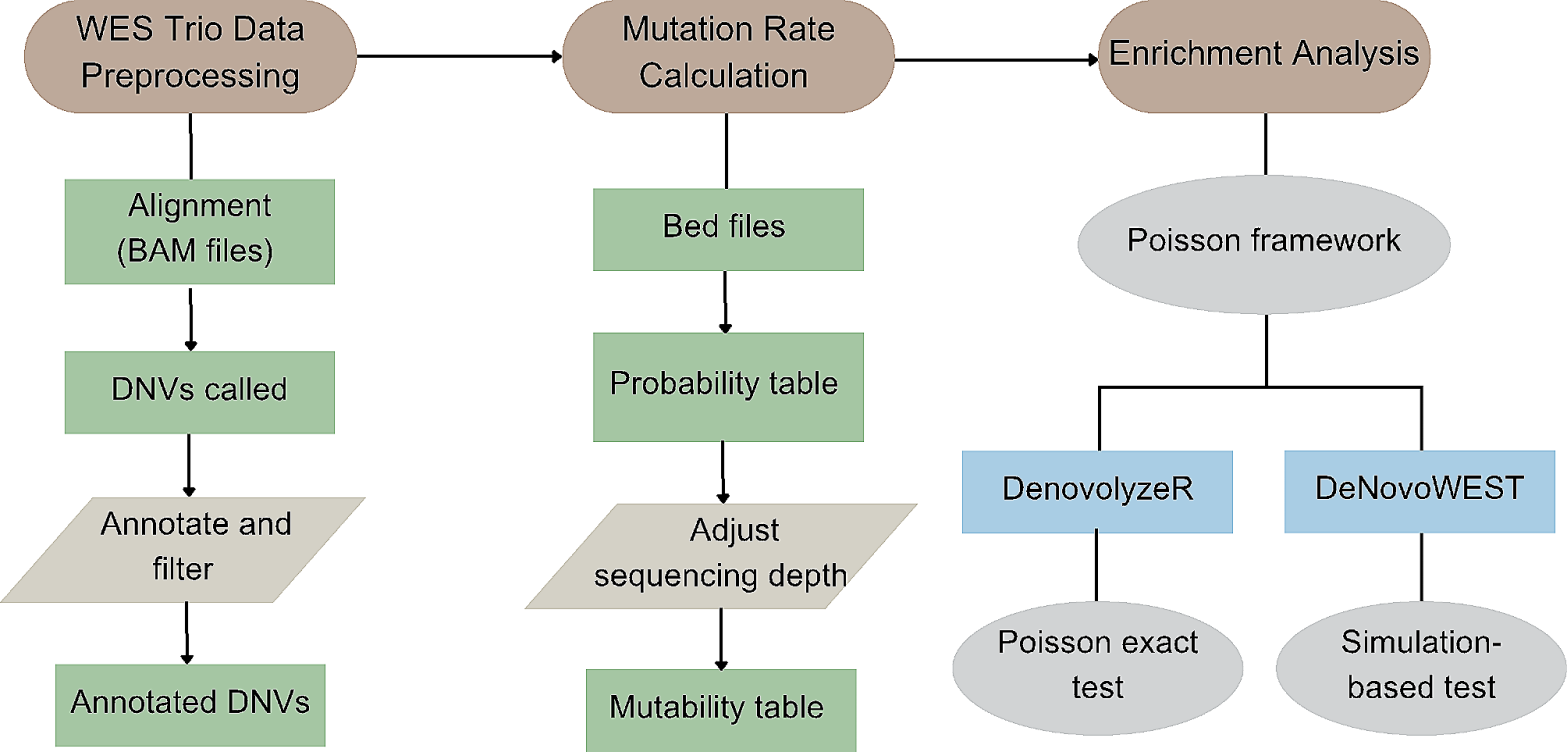
General workflow of DNV analysis. In step 1, sequencing data from trios are preprocessed and annotated. In step 2, mutation rates are calculated for each gene. In step 3, enrichment analysis is conducted to identify risk genes

In this section, we summarize the general workflow of DNV analysis into three steps: data preprocessing, mutation rate calculation, and enrichment analysis. An illustration of the general workflow from step 1 to step 3 is shown in Fig. 1.

### Step 1: data preprocessing

After samples are sequenced via WES or WGS, DNVs need to be called from unmapped sequencing reads. In 2021, Diab et al. published a detailed protocol for analyzing germline DNVs from WES [21]. Briefly, two steps are needed before trio DNV analysis. In the first step, binary alignment/map (BAM) files are generated from unmapped sequencing reads. In the second step, variants are called based on the GATK best practices [22]. Compared with other types of variant calling, DNVs are required to be jointly called in trios, where pedigree files for trios will be generated. For each trio, VCF files are generated based on GATK best practices and further processed, including steps such as splitting multi-allelic sites and left normalization by BCFtools [23]. Then, the generated variants are annotated and filtered based on minor allele frequencies and alternate allele ratios in probands and parents. After the above analyses, all candidate DNV calls are manually verified using the integrative genomics viewer (IGV) [24]. Before proceeding with enrichment analysis, variants are further classified into loss of function (LoF), damaging missense (Dmis), and other groups using annotation tools such as ANNOVAR [25, 26]. Step-by-step procedures can be referred to in the original protocol [21].

### Step 2: mutation rate calculation

After variant calling is completed, the next step is to estimate the per gene mutation rate. In 2012, Neale et al. [27] developed a statistical model for estimating the expected mutation rate in the exome. They assess the mutation rates of all possible trinucleotide contexts within the intergenic region of the human genome. They considered variations in two ways: comparing the fixed genomic difference to chimpanzees and baboons [28], and variations identified through the 1000 Genomes Project [29]. The mutation rate for the exome was estimated by summing up the mutation rates for all bases captured by the exome, and that of each functional annotation class was determined by summing the mutation rates of variants belonging to that class.

In 2014, Samocha et al. extended Neal’s framework to calculate each gene’s expected rate for different types of mutation [30]. There are two steps of the framework. First, the sequence context is used to estimate the probability of each base mutating to another base. Second, based on the change of trinucleotide, the outcome of each type of base change is identified, including synonymous, missense, nonsense, essential splice site and frameshift mutations. These probabilities are added up to obtain a mutation rate per gene for different types of mutations. The input includes bed files where each row represents a specific genomic region from exome capture, trio information, and sequencing coverage of WES samples that can be calculated using Mosdepth [31]. More specifically, bed files should be first converted into sequence data that contain four base information (ATCG) and subsequently transformed into a probability table. Then, annotations are added to the table using ANNOVAR. Next, the per-base mutation rate is adjusted by sequencing depth. For each base, the number of trios with 10x or greater coverage is counted. The numbers are adjusted with a coefficient ranging from 0.9 to 1, which assigns greater weights to bases with higher sequence depths.

### Step 3: enrichment analysis of DNVs

Enrichment analysis of DNVs aims to find elevated or excessive gene burden due to DNVs. The goal is to assess whether there is a significant accumulation or enrichment of DNVs within specific genes. Statistical methods for enrichment analysis test whether the observed DNVs occur more frequently than expected by chance in a gene.

Current methods for DNV enrichment analysis are mostly developed based on a Poisson framework, which assumes the number of observed DNV counts follows a Poisson distribution. Some methods focus only on DNVs, and others can incorporate information from other types of variants or other biological information. Among methods that focus on DNVs, Ware et al. proposed one of the first models based on the statistical framework of Samocha et al. to analyze coding DNVs and named the R package of the model DenovolyzeR [8, 30]. The framework assumes the number of observed DNV counts in a single gene ($m$) follows a Poisson distribution, and a certain type of variants (e.g. LoF) with the expected counts calculated as two times the product of sample size and mutation rate of the corresponding type. It uses a Poisson exact test to compare the observed counts of the type of variants with the expected counts:

$$m\sim\text{P}\text{o}\text{i}\text{s}\text{s}\text{o}\text{n}\left(\lambda \right),$$

where $\lambda$ is the mean of the distribution.

DenovolyzeR provides a pre-calculated mutability table from Samocha et al. that can be used to conduct four types of enrichment analyses [8, 30]. These include (1) assessment of genome-wide burden of different types of DNVs, (2) assessment of burden of genes with multiple DNVs, (3) assessment of whether a single gene carries an excess number of mutations, and (4) assessment of whether a gene set is enriched with DNVs. Details of the analysis step can be found in the original protocol [8].

DeNovoWEST (*De Novo* Weighted Enrichment Simulation Test) is another method proposed for DNV enrichment analysis that uses a simulation-based statistical test to detect gene-specific enrichment of DNVs [32]. It includes two components: an overall enrichment test that includes all nonsynonymous DNVs and a clustering test that assesses the enrichment of missense variants. The overall enrichment test is a simulation-based test that calculates the probability of observing the severity of a gene higher than expected, considering all possible numbers of DNVs per gene:

$$P\left(S\ge s\right)\approx \sum _{k=0}^{250}P(S\ge s|k\left)P\right(K=k)$$,

where $S$ denotes the gene severity score, $s$ denotes the observed severity score, and $K$ is the number of DNVs in the gene. The severity score of each DNV is an empirically estimated positive predictive value of being pathogenic based on its predicted protein consequence, CADD score, and selective constraint against heterozygous protein truncating variants in the gene, and it is calculated by summing the severity scores of mutations within this gene. The upper limit of k is chosen as 250 as this number is far larger than the observed DNV counts in real data. When $k=\text{0,1}$, $P(S\ge s|k\left)P\right(K=k)$ can be calculated analytically. When $k\ge 2$, $P(K=k)$ can be calculated analytically under the null assumption that DNVs follow a Poisson distribution, and $P(S\ge s|k)$ can be estimated using a simulation-based approach. The estimated value of the probability $P\left(S\ge s\right)$is defined as *pEnrich*.

The missense enrichment test is performed the same as the overall enrichment test, except that only missense variants are included in the score calculation. The proportion of the simulated scores for missense variants that are larger than the observed scores is defined as *pMisEnrich*. For the clustering test, the clustering distance is determined as the geometric mean coding distance between all potential pairs of DNVs [33]. The observed distance of missense DNVs is then compared with the expected distance from simulated missense DNVs, and the probability of missense variants being as or more clustered than the null model is defined as *pClustering* [34]. *pMisEnrich* and *pClustering* are then combined using Fisher’s method to obtain *pMEC*.

*pMEC* = combined (*pMisEnrich*, *pClustering*).

The final testing *p*-values for *DeNovoWest* are obtained by taking the minimum of *pEnrich* and *pMEC*. Bonferroni correction is used to account for multiple testing comparisons.

*pDeNovoWEST* = min(*pEnrich*, *pMEC*).

## Integrative analysis of DNVs and other types of variants

Studies on DNVs often lack statistical power due to their relatively low frequency. Efforts have been made to increase statistical power by incorporating other biological information.

To integrate other types of variants with DNVs, He et al. proposed a hierarchical Bayesian framework named the transmission and *de novo* association (TADA) test to incorporate the information from both inherited and DNVs [17]. Assuming subjects can be classified as carrying two alleles, TADA denotes alleles with deleterious mutation as *a*, and alleles without as *A*, and $\gamma$ denotes the relative risk of *Aa* compared with *AA*. TADA tests the null hypothesis of $\gamma =1$ against the alternative hypothesis of $\gamma \ne 1$ for all genes. TADA has gained success in its application in multiple studies [35–39]. However, it requires external information or prior knowledge to estimate hyperparameters. In 2017, Nguyen et al. adopted a fully Bayesian framework to extend TADA and named their method extTADA [40]. However, both TADA and extTADA do not consider the recessive mode of inheritance in their models.

In 2021, Li et al. proposed a TADA-R model built upon TADA to include the recessive disease model, including both the cases of homozygotes and compound heterozygotes [41]. By applying TADA-R to CHD, they identified 15 significant genes, many of which were not implicated in previously published studies.

Multiple methods have been proposed to incorporate functional annotation to boost the statistical power of risk gene identification. fitDNM [42] is a method that tests the excess *de novo* load of genes by deriving score statistics from a retrospective likelihood that incorporates functional information quantitatively rather than classifies variants into different functional categories like TADA. They integrated the probabilities of a mutation functionally impacting the gene when characterizing the distribution of DNVs in an affected individual and estimated these probabilities using scores that are predicted externally. Specifically, they defined the probability that the mutation is damaging to protein function when characterizing the distribution of DNVs at a locus, and set the probability of LoF variants predicted by SnpEff [43] as 1, the probability of missense variants as their PolyPhen-2 [28] scores, and that of synonymous variants as 0. It was observed that fitDNM had increased power compared to Poisson tests and TADA tests with DNVs only in the simulation studies and real data analyses.

Similar to fitDNM, Hu et al. developed a likelihood ratio test named VARPRISM that incorporates variant prioritization to test associations of DNVs [44]. Although VARPRISM shares a few features with fitDNM, it utilizes different strategies to incorporate functional information. VARPRISM employs the likelihood ratio of a variant being damaging versus neural by incorporating a conservation-controlled amino acid substitution matrix (CASM) from VAAST 2.0 [45], while fitDNM requires probabilities of given mutations being damaging from an external resource instead of being estimated directly from data. Hu et al. showed that VARPRISM had better power than fitDNM with two simulated datasets.

Efforts have also been made to integrate information from noncoding regions. TADA-A is a statistical framework that models mutation counts for each position in the genome with Poisson distribution [46]. The model can combine genomic annotation information from both coding and non-coding regions. Furthermore, TADA-A supports meta-analysis of multiple DNV studies by fitting a background mutation model for each study to adjust for potential technical factors. However, TADA-A only focuses on regulatory sequences close to genes without considering those distal to transcription start sites. Also, it uses a linear model to combine information from different annotations, which may not be as powerful as using a non-linear model such as deep neural networks [46, 47].

HeartENN (Heart Effect Neural Network) is developed to identify noncoding DNV burden in CHD. It is extended from a deep learning-based framework for predicting the effects of non-coding variants named DeepSEA [48, 49]. HeartENN uses two neural network-based epigenomic models for human and mouse to predict genome-wide features based on heart-specific chromatin profiles. It was found that noncoding variants prioritized by HeartENN score (score ≥ 0.1) had significant enrichment of known human CHD genes in CHD cases.

## Integrative analysis of DNVs and other sources of biological information

### Muti-trait methods

Extended from extTADA, Nguyen et al. developed a multi-trait Bayesian framework that can jointly analyze two traits named mTADA [50]. There are four hypotheses of the model: the gene is associated with neither trait (${H}_{0}$), the gene is only associated with the first trait (${H}_{1}$), the gene is only associated with the second trait (${H}_{2}$), and the gene is associated with both traits (${H}_{3}$). The input data includes DNVs from the two cohorts and mutation rates of genes that can be calculated from an external framework. The mTADA framework assumes the DNV counts for both traits follow Poisson distributions. When gene *i* is associated with trait $k$ (*k* = 1 or 2), the rate parameter in the Poisson distribution of its DNV count is multiplied by a relative risk parameter ${\gamma }_{ik}$. ${\gamma }_{ik}$ is assumed to follow a Gamma distribution with two parameters ${\stackrel{-}{\gamma }}_{k}$ (mean relative risk) and ${\beta }_{k}$ (to control the variance of the relative risk). Based on the four hypotheses, the corresponding posterior probabilities for genes can be calculated from Markov chain Monte Carlo. However, the hyperpriors of mTADA cannot be estimated from the data but rely on running extTADA first.

M-DATA is another multi-trait method that shares the same hypotheses as mTADA, but uses an Expectation-Maximization algorithm to estimate parameters and infer risk gene status [51]. Compared to mTADA, this method uses an alternative way to characterize the effects of variants in different functional groups by linking variant-level and gene-level functional annotations to the relative risk of *de novo* genotype in the model. However, M-DATA requires users to preselect functional annotations before inputting the data and the algorithm cannot prioritize the functional annotations automatically. In addition, if the underlying functional annotation effect size is small, the power improvement of M-DATA compared with models without integrating functional annotations will be minor.

Quantifying the genetic association of DNVs between different genetic disorders is crucial because it can lead to a better understanding of the common molecular foundations these disorders may share. While recent research has shown that certain genes and biological pathways are commonly affected by DNVs in various disorders, current methods tend to only consider genes that are statistically significant across multiple disorders and cannot fully capture the complexity of genetic associations due to the polygenic nature of diseases and incomplete penetrance. EncoreDNM is a novel statistical method that quantifies the overall genetic sharing of DNVs between two disorders for different variant types [52]. Instead of using the Bayesian framework, it constructs mixed-effects Poisson regression models to evaluate the correlation between two traits by providing the estimated correlation and *p*-values from statistical inference. This method is designed for testing global genetic architecture from DNV information across traits but does not provide a way to prioritize specific risk genes.

### Network-assisted models

Risk genes identified from DNV studies have been shown to enrich in a protein-protein interaction (PPI) network in the post-association analysis [50]. Two methods have been proposed to integrate network information with DNV data based on the assumption that neighboring genes are more likely to have similar disease association statuses.

DAWN is a post-association method that takes association results from TADA *p*-values and gene-gene interaction network estimated from expression data as input [53, 54]. In real data application, Liu et al. identified 333 genes that plausibly affect autism risk by integrating association results from WES data and brain gene expression data [54].

Compared to DAWN, N-DATA is a model that does not require summary statistics results from other methods such as TADA [55]. It directly incorporates PPI information into the prior risk gene status based on the Poisson mixture distribution. After applying N-DATA to real DNV data from the CHD study, Xie et al. identified 46 candidate genes with at least one DNV in the study cohort. Among these genes, they discovered that some genes can only be identified after integrating the network information compared with existing genes that can be identified using the baseline model without integrating the network. Visualizing the 46 genes in the PPI network, they found three main gene clusters formed that are biologically interpretable within the network, including one cluster related to transcriptional regulation and early cell growth or differentiation processes, one cluster related to RNA splicing, and the third cluster related to protein synthesis. This further demonstrates the improvement of power after incorporating network information into the framework. In simulation studies, Xie et al. showed that the performance of N-DATA and DAWN was comparable when the signals in the network became stronger. However, with more and more network databases available, these methods did not provide a way to incorporate multiple types of interactions or to prioritize network information.

### Integrative analysis of DNVs and expression data

VBASS is an integration model that incorporates bulk or single-cell expression data into the analysis of DNVs based on a Bayesian framework to discover risk genes [56]. It constructs a model of disease risk based on expression profiles, which are estimated using deep neural networks. It simultaneously trains the neural network weights and determines the parameters for the Gamma-Poisson likelihood model of DNV counts based on both gene expression data and genetic data. Different from previous methods, it has the key assumption that the prior probability of a gene being a risk candidate should be specific at the gene level and could be inferred from gene expression information in relevant tissues. Therefore, it takes the gene expression profiles as a vector into its probabilistic model and estimates parameters in the model using deep neural networks. In addition, it can also incorporate RNA sequencing data of relevant organs or cell types other than single-cell expression profiles. The performance of VBASS is highly dependent on the quality of the gene expression data. One practical issue that may hamper incorporating gene expression information with DNVs in birth defect studies is that gene expression data of early developmental human organs are hard to acquire.

## Future directions

### Sex-aware models

There is accumulating evidence of sex bias in neurodevelopmental disorders. For instance, the diagnosed male-to-female ratio of autism spectrum disorder (ASD) is three to four times. The female protective effect can be attributed to genetic, hormonal, and environmental factors. Limited studies have focused on studying the sex-based mechanisms related to DNVs.

In 2017, Turner et al. conducted sex-based enrichment DNV analysis for neurodevelopmental disorders using ∼ 8,825 sequenced parent-offspring trios in denovo-db as the discovery cohort [57, 58]. The discovery cohort identified 17 female-only significant genes, 18 male-only significant genes, and 19 shared significant genes. Among the 54 genes identified, 25 genes were replicated in the 18,778 trios from the GeneDx cohort [58]. They not only observed significant enrichment on the X chromosome for females but identified potential sex-biased genes on autosomal chromosomes.

Similar to ASD, the prevalence and disease mechanism of birth defects can vary based on sex. For instance, a review study surveyed 21 articles and confirmed a significant gender variation in specific CHD subgroups [59]. These findings call for more DNV studies to test sex-based enrichment in birth defects and suggest the potential to develop methods that can incorporate sex-specific mechanisms.

### Integration of common variants with DNVs

Common variants have been shown to play an important role in complex human traits and diseases. Despite the success of identifying risk genes using rare variants such as DNVs, it is worth exploring the joint and different contributions of both rare variants and common variants. Different hypotheses have been made about the contribution of common and rare variants in complex human diseases. In a study on UK Biobank participants, Lu et al. found that rare pathogenic variants were more prevalent among patients with a low polygenic risk score (PRS) affected by diseases including breast cancer, colorectal cancer, type 2 diabetes, osteoporosis, and short stature [60]. Studies in ASD have also suggested that DNVs and common variants have addictive effects [61, 62]. Another study has suggested that genes harboring schizophrenia-associated common variants and genes harboring DNVs both contribute to a core set of biologically important pathways and networks and the interactions of these genes may play a part in the risk of schizophrenia [63]. To better prioritize suggestive loci from OFC GWAS, Bishop et al. hypothesized DNVs near a GWAS peak could provide evidence in support of suggestive loci without reaching formal significance [9]. After evaluating genes within 1 Mb (± 500 kb) of both suggestive and significant loci from two recent OFC GWAS studies, they found 37 protein-altering DNVs were within these genes and several of them were located in genes implicating OFC development. These results suggest a potential future direction in integrating common variants and DNVs in a general framework for birth defects.

### Integration of epigenetic information with DNVs

Epigenetic changes modify the activation of certain genes without changing the DNA sequence, and they play an essential role in human development and disease etiology [64]. There are different classes of epigenetic information including DNA methylation, histone modification, and noncoding RNA action [65]. DNVs and epigenetic interactions may interact to influence gene expression and contribute to disease development and progression [66]. For instance, DNV in the *RING1* gene was identified in a 13-year-old girl with neurodevelopmental disabilities. *RING1* encodes an E3-ubiquitin ligase that is involved in the epigenetic control of transcription during development. The mutant *RING1* retained catalytic activity but was unable to ubiquitylate histone H2A. The animal model suggested that animals with the same mutation or complete knockout of *RING1* ortholog had defects in histone H2A ubiquitylation. *RING1* mutations are likely a cause of human neurodevelopmental disorders where epigenetic effects play an important role [67].

Epigenetic regulation mechanisms have also been identified for CHD. For instance, researchers conducted a case-control study using exome sequencing to compare the occurrence of DNVs in genes related to the modification of histone proteins in individuals with severe CHD and those without the condition. They discovered an excess of DNVs in the genes responsible for writing, erasing, or reading H3K4 methylation or H2BK120 ubiquitination required for H3K4 methylation. This indicates a potential pathogenic role of abnormal histone methylation in CHD [11, 68].

Studies have also shown that integrating genetic data with epigenetic information could better elucidate functional insights of complex diseases [69–71]. For example, Andrews et al. found ASD-associated SNPs in GWAS are enriched for tissue-specific meQTLs in fetal brain and peripheral blood [70].

These studies collectively demonstrate the potential of integrating epigenetic information with DNVs to understand the complex mechanisms underlying birth defects.

### Integration of protein structural information with DNVs

Protein Data Bank (PDB) is a worldwide repository that stores 3D structural information about biological macromolecules. Despite the efforts in experiments, only 35% of human proteins are mapped to a PDB entry, and frequently, these entries represent only fragments or segments of protein sequences rather than the whole proteins [72, 73]. The release of AlphaFold2 expands the coverage of human protein structures to 98.5%, with 58% of them with high confidence [73]. The enlarged knowledge of protein structures can help us elucidate the molecular mechanisms of more variants. Leveraging protein structures from protein structure databases, three statistical methods - POINT [74], PSCAN [75], and POKEMON [76] - have been developed to characterize the association between rare missense variants and phenotypes by integrating 3D spatial distance of variants within protein structures.

In addition to spatial distance, studies have also shown the importance of functional features, physiochemical features, interaction features, and others. Multiple studies showed differences of these features between pathogenic missense variants and variants that are commonly found in the general population [77–79]. For instance, Iqbal et al. surveyed 40 structural features and found significant enrichment of multiple features under different categories in pathogenic missense variants from Clinvar [80] and HGMD [81] compared with general population variants from gnomAD [78, 82] in 1,330 disease-associated genes. Chen et al. demonstrated that ASD cases exhibited an enrichment of *de novo* missense variants with disruptive impacts on protein interactions, and these variants often affect hub proteins and disturb their interactions [83]. These findings suggest the potential benefit of new methods that can integrate different categories of protein structural features such as secondary structures, residue exposure levels, and PPIs into the modeling of *de novo* missense variants.

## Conclusions

In this review, we have summarized different statistical methods that can be applied to identify risk genes for birth defects by analyzing DNVs. Most of the methods characterize DNV counts within a gene using a Poisson distribution and estimate parameters using likelihood-based approaches or Bayesian methods. The output of these methods includes *p*-values, posterior probabilities, Bayesian *q*-values, and others in correspondence to different modeling approaches. The identified risk genes by these methods can help guide future biological experiments and clinical studies to further understand disease mechanisms. In the meantime, the complexity of genetic architectures, the interplay between genetic and environmental factors, and the rare nature of DNVs pose challenges to the current field. Moreover, the methods that can integrate multi-layers of multi-omics data, such as integrating transcription and methylation data with DNVs remains in the early stage of development. There is also a critical need for improved computational models that can effectively incorporate the heterogeneity of birth defects, and for databases that can capture the phenotypic spectrum associated with DNVs in a standardized way. Addressing these challenges requires interdisciplinary collaboration and the development of innovative analytical tools capable of dissecting the intricate biological networks underlying birth defects. To better elucidate the etiology of birth defects, we discussed several potential future directions, including incorporating information on sex, common variants, epigenetic information, and protein structures with DNVs. These future directions offer abundant possibilities, inviting researchers to unlock the mysteries of genetic etiology and developmental biology for birth defects and paving the way for personalized therapeutic strategies.

## Data Availability

Not applicable.

## Abbreviations

WES: Whole exome sequencing
WGS: Whole genome sequencing
SNV: Single nucleotide variant
DNV: *De novo* variant
CHD: Congenital heart disease
CDH: Congenital diaphragmatic hernia
OFC: Orofacial cleft
FDR: False discovery rate
LoF: Loss of function
Dmis: Deleterious missense
IGV: Integrative genomics viewer
PPI: Protein-protein interaction
ASD: Autism spectrum disorder
PRS: Polygenic risk score
PDB: Protein Data Bank
